# Corrections required for Dressler and Johnson 2022

**DOI:** 10.1007/s00702-022-02534-0

**Published:** 2022-08-30

**Authors:** Alberto Esquenazi, Mark Elliott, Andreas Lysandropoulos

**Affiliations:** 1 MossRehab & Albert Einstein Medical Center, 60 Township Line Rd., Elkins Park, PA 19027 USA; 2 Ipsen Bioinnovation, Abingdon, UK; 3 Ipsen, Boulogne-Billancourt, France

Dear Editors,

We read with interest the recent published review by Dressler and Johnson (2022) and would like to offer some important updates and corrections to some of the statements made to avoid misconceptions that unfortunately persist to this day from previous work (Pickett 2011).

First, we must clarify Ipsen’s cell-based assay which is fully implemented for abobotulinumtoxinA product supplied to the European Union, United States (US) and Canada, and that these important changes were well publicized in 2019 and 2020, respectively (Ipsen 2019, 2020a). The US prescribing information notes that the *“…primary release procedure for DYSPORT uses a cell-based potency assay to determine the potency relative to a reference standard”* (Ipsen 2020b). In addition, several other regions and countries have since added to the list without press release.

Second, we cannot condone any intimation that certain botulinum toxin products are comparable. Specifically, we are concerned that by saying abobotulinumtoxinA has *“idiosyncratic potency labelling”*, the reader may mistakenly infer that other available botulinum toxin type A products (e.g., onabotulinumtoxinA and incobotulinumtoxinA) are comparable—when there is no consensus on this. In fact, regulatory agencies in the US, Europe, and most other countries worldwide require a statement that botulinum toxin units are not interchangeable from one product to another. This reflects the fact that these are biological products and, as Dressler and Johnson highlight in their review, have clear differences in the manufacturing processes, formulations, and assay methods used to determine units of biological activity. Furthermore, each product may behave differently depending on where it is targeted (Brin et al. 2014).

While conversion ratios abound in the literature, the practical implications of non-interchangeability of current botulinum toxin products are crucial to understand, because it means that the risk–benefit ratio for each of the products (including efficacy, safety, immunogenicity, and duration of action) must always be considered individually. In the absence of well-designed comparative studies, the best way to do this is to consider the evidence of safety and efficacy for each product based on their indication. Our stance will almost certainly change in the future when recombinant technologies become clinically available (Fonfria et al. 2018) and potency units obsolete.

Respectfully

Alberto Esquenazi, Mark Elliott, Andreas Lysandropoulos
